# Creation of unexplored tunnel junction by heterogeneous integration of InGaAs nanowires on germanium

**DOI:** 10.1038/s41598-022-05721-x

**Published:** 2022-01-31

**Authors:** Akinobu Yoshida, Hironori Gamo, Junichi Motohisa, Katsuhiro Tomioka

**Affiliations:** 1 Graduate School of Information Science and Technology, Hokkaido University, North 14 West 9, Sapporo, 060-0814 Japan; 2 Research Center for Integrated Quantum Electronics (RCIQE), Hokkaido University, North 13 West 8, Sapporo, 060-0813 Japan

**Keywords:** Structural materials, Nanoscale materials, Electronic properties and materials, Nanowires, Synthesis and processing, Materials science, Electronic devices

## Abstract

Heteroepitaxy has inherent concerns regarding crystal defects originated from differences in lattice constant, thermal expansion coefficient, and crystal structure. The selection of III–V materials on group IV materials that can avoid these issues has however been limited for applications such as photonics, electronics, and photovoltaics. Here, we studied nanometer-scale direct integration of InGaAs nanowires (NWs) on Ge in terms of heterogenous integration and creation of functional materials with an as yet unexplored heterostructure. We revealed that changing the initial Ge into a (111)B-polar surce anabled vertical InGaAs NWs to be integrated for all In compositions examined. Moreover, the growth naturally formed a tunnel junction across the InGaAs/Ge interface that showed a rectification property with a huge current density of several kAcm^−2^ and negative differential resistance with a peak-to-valley current ratio of 2.8. The described approach expands the range of material combinations for high-performance transistors, tandem solar cells, and three-dimensional integrations.

## Introduction

As the progress of epitaxy has led to the innovation of solid-state lighting technology^1–6^, innovation in semiconductor devices and progress in epitaxial growth have become inseparable. This is true not only in the regard to the development of optical devices but also that of electronic devices such as high mobility transistors^7, 8^. Once the technology to stably grow high-quality crystals in unexplored materials that can meet increasingly sophisticated requirements for high performance and high functionality that surpass conventional materials and compatibility of conflicting functions, has been established, innovations have been created. In this regard, we focused heterogeneous integration of InGaAs and Ge for future electronics and photonics such as hybridized InGaAs/Ge complementary metal–oxide–semiconductor (CMOS) applications and multijunction tandem solar cells^9–13^.

For CMOS applications, the primary challenge is to reduce power consumption while enhancing performance. Here, InGaAs and Ge are alternative channel materials because of their high carrier mobility compared with Si. In particular, In-rich InGaAs (with an In composition of about 53–80%) would be a good choice for next-generation n-type field-effect transistors (FETs) because of its electrostatic gate controllability^14^. Moreover, InGaAs nanowires (NWs) with Ge overcome mobility mismatch of InGaAs and Ge in CMOS integration with current matching. The mobility mismatch emerges as miniaturization progresses. As the MOSFETs is reaching to several nanometer-scale, the miniaturization could be a bottleneck in shrinking III–V and Ge MOSFETs because there is as yet no effective approach to integrate these channel materials on Si platforms at this scaling level. A hybrid logic architecture using a vertical III–V nanowire (NW) channel directly integrated on a p-type Ge MOSFET as a vertical gate-all-around (VGAA) structure could be a way to shrink the effective device area compared with a planar architecture and enable equivalently down-scaled n-type MOSFETs^15^.

The material combination of the InGaAs/Ge had advantages for multijunction tandem solar cell toward high energy-conversion efficiency^13^. Here, buffer technology, such as a step graded layer, is used to avoid lattice relaxation that degrades the performance of photovoltaic devices because of misfit dislocations. The use of NW structures can decrease the density of dislocations at heterojunction because their nanometre-scale cross-section effectively suppresses their formation of misfit dislocation remaining lattice strain. Thus, Ga-rich InGaAs NWs (In composition of 10–30%) integrated with Ge would be able to form a dislocation-free tunnel junction composed of InGaAs and Ge without the buffer or graded layer.

In this report, we demonstrate heterogeneous integration of InGaAs NWs with various In compositions and examine the controllability of the NWs’ composition for electronic and optical applications. The composition of In atoms in the NW solid phase was found to be higher than that in vapour phase at the optimum growth temperature (*T*_G_) because the optimum *T*_G_ for the NW growth was dominant for the desorption process and Ga adatoms desorbed more easily than In adatoms. Transmission electron microscopy (TEM) revealed that InGaAs NW epitaxially grew on Ge, but the InGaAs layer diffused into the Ge substrate to a depth of several nanometres from the interface. The vertical NW diode using the vertical InGaAs NW/Ge exhibited rectifying properties with negative differential resistance (NDR), indicating band-to-band tunnelling although the InGaAs/Ge was not heavily doped. This indicated that the Group-III atoms diffused into the Ge substrate and the Ge atoms simultaneously outer diffused into the InGaAs NWs. Thus, heavily doped n-InGaAs and p-Ge layers were formed across the InGaAs/Ge junction.

## Results

### Heterogeneous integration of vertical InGaAs NWs on Ge

The growth morphology of selective-area epitaxy of In_x_Ga_1-x_As NWs on Ge(111) was characterized at various growth temperatures (*T*) and In contents in the vapour phase. Moreover, the controllability of the growth direction of the NWs was evaluated under these growth conditions. The optimum *T* (*T*_G_) for selective-area epitaxy of InGaAs NWs was varied with In compositions in the vapour phase, because *T*_G_ has been found to be 540 °C for InAs NWs and 700–750 °C for GaAs NWs^16–18^. Moreover, the method of aligning vertical III–V NWs on Ge(111) was different between the GaAs and InAs NWs although modifying the Ge(111) to have a (111)B-polar nature at low temperature annealing was common^19, 20^. In particular, low-temperature buffer layer growth was effective for aligning vertical GaAs NWs^14^, while pulsed growth mode was effective for aligning vertical InAs and InGaAs NWs^15, 20^.

**Figure 1:**
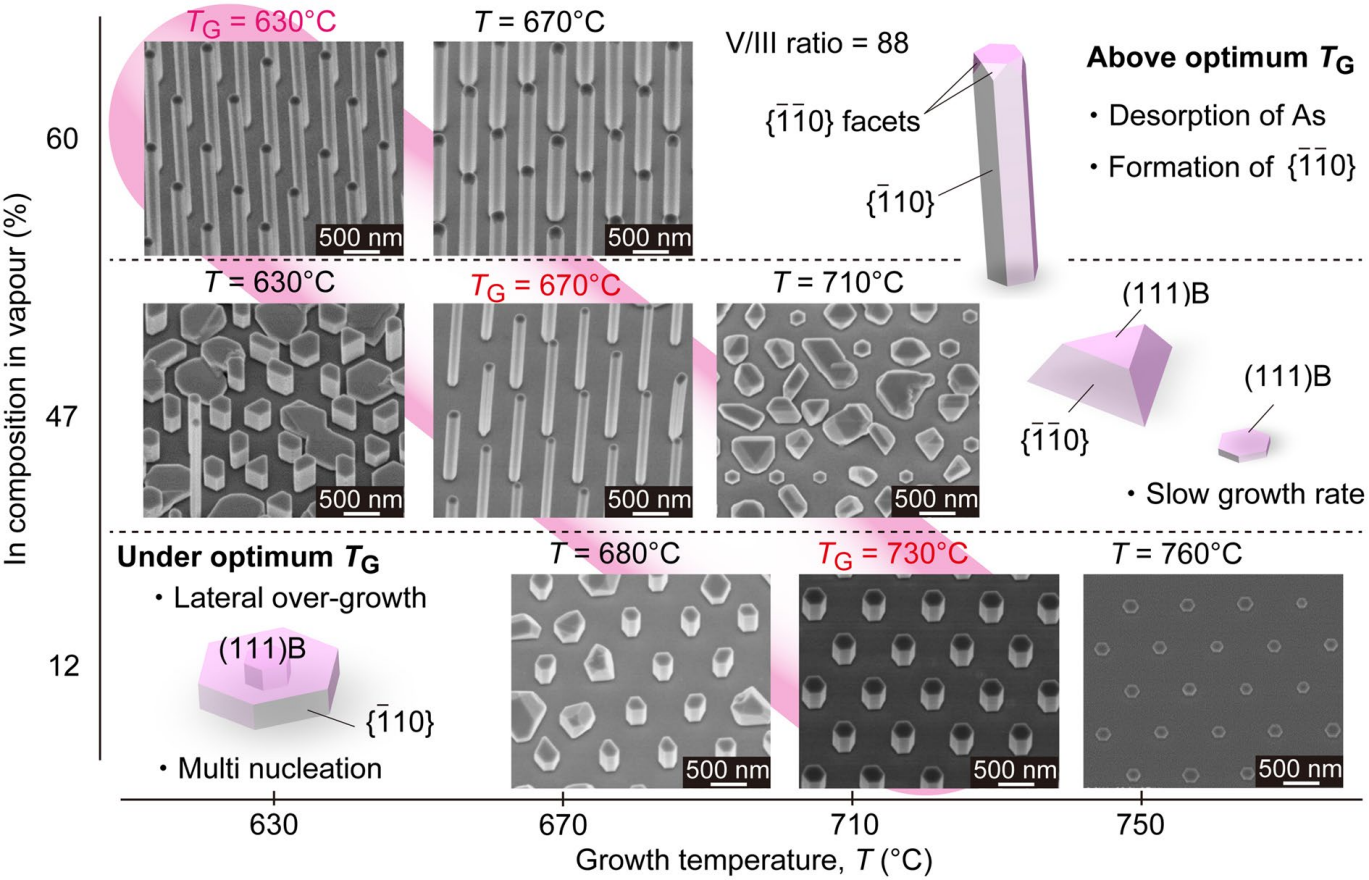
Selective-area growth of InGaAs on Ge(111). Growth morphologies of InxGa1 − xAs NWs on Ge(111) at various growth temperatures (T) and In compositions in vapour phase (x).

Figure 1 summarizes the growth morphologies of the InGaAs NWs grown at various growth temperature (*T*) and In contents in the vapour phase. Here, the optimum *T*_G_ (highlighted in pink in Fig. 1) is defined as the value yielding a moderately suppressed growth rate along lateral <−110> direction due to low As coverage with less adsorption of III atoms on the {− 110} side facets and a the NW diameter close to the opening diameter. It varied from 630 to 730 °C with the In composition. Pulsed growth mode was used in all conditions, and it had a strong effect on aligning the NW’s growth in the vertical [111] direction. This results indicate (111)B-polar surface efficiently formed.

For 47% In in the vapour phase, the optimum *T*_G_ for the vertical InGaAs NWs was at around 670 °C (middle images of Fig. 1). At *T*_G_ (in this case *T* = 630 °C), lateral over-growth in the <  − 110 > directions was enhanced (bottom left in Fig. 1). The InGaAs coalesced with each other (middle left image of Fig. 1) because the As coverage was enhanced on the {− 110} facet and the crystallization became faster because of the large number of In and Ga adatoms. The growth rate in the vertical <111> B direction was suppressed by the formation of As trimers^20^. Above the optimum *T*_G_ (710 °C in this case), hillocks covered with {−1–10} facets appeared and short hexagonal pillars formed (middle right image in Fig. 1). Their appearance was due to desorption of group-III and As atoms at the (111)B plane. The surface diffusion length of the In/Ga atoms was shortened and desorption process increased at the edge between the (111)B and vertical {− 110} facets, a phenomenon that has been observed in selective-area growth of AlGaAs^21,22^.

For 60% In content in the vapour phase, the optimum *T*_G_ for vertical NWs was at around 630 °C (upper left image in Fig. 1). Above the optimum *T*_G_, the NW morphology had {−1–10} facets on top of the NWs (upper right image in Fig. 1). The mechanism of forming the {−1–10} facets was the same as in the case of 47% In content.

For 12% In content in the vapour phase, the optimum *T*_G_ for vertical NWs was at around 730 °C (bottom images in Fig. 1). However, a slight amount of the lateral over-growth occurred and there was no *T*_G_ that completely suppressed the lateral over-growth. This assumed that the pulsed growth mode under Ga-rich conditions enhanced lateral over-growth in the early stage of InGaAs growth and the InGaAs overflowed around SiN mask^20^. Consequently, the grown InGaAs NWs had a larger diameter than that of the openings. Below *T*_G_, hillock structures that rose from the non-conformal lateral over-growth formed through a multi-nucleation process in the early stage of the InGaAs growth, which is a similar to the finding in a previous report^20^. Above *T*_G_, short hexagonal pillars formed because the enhanced desorption shortened the diffusion length of the supplied source atoms and increased desorption process on (111)B surface. Overall, the growth morphologies shown in Fig. 1 indicated that the optimum *T*_G_ depended on the In content in the vapour phase and the *T*_G_ window and their growth morphologies were the same as in the conventional selective-area growth of InGaAs NWs once the Ge initial surfaces were converted into (111)B-polar surfaces.

**Figure 2:**
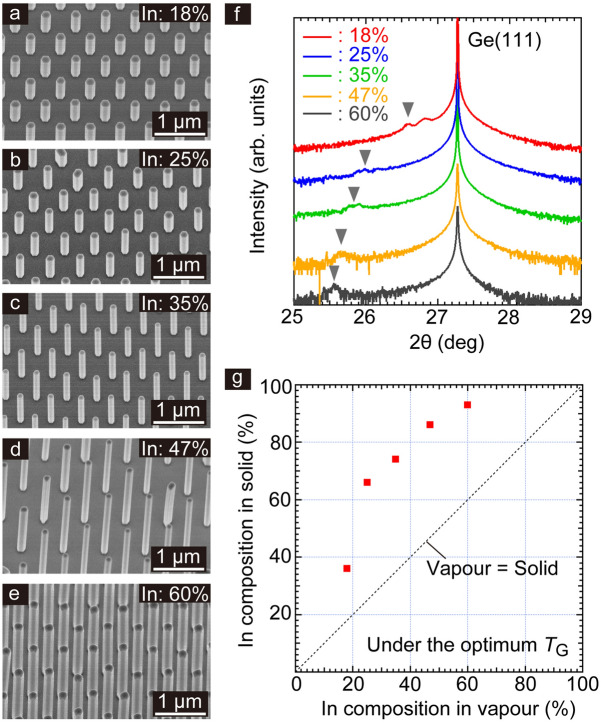
Direct integration of vertical InxGa1 − xAs NWs on Ge(111). 30°-tilted SEM images showing InGaAs NWs on Ge that were grown with various In compositions in vapour phase (**a**) 18%, (**b**) 25%, (**c**) 35%, (**d**) 47%, and (**e**) 60%. The NWs were grown at *T*_G_. (**f**) XRD spectra of InGaAs NWs on Ge in (**a**–**e**). (**g**) In composition in the solid phase of InGaAs NWs as estimated from the (**f**). The dashed line shows that the In composition in the vapour phase is equal to that in the solid phase.

Figure 2a–e show representative NW-growth results for various In-compositions in the vapour phase at *T*_G_. The (111)B-polar surface virtually aligned the vertical InGaAs NWs. As following the growth behaviour in Fig. 1, the InGaAs NWs in Fig. 2a were grown at 730 °C. The NWs in Fig. 2b–d were grown at 670 °C. The NWs in Fig. 2e were grown at 630 °C. The composition of In in the solid phase of the InGaAs NWs was characterized by XRD (Fig. 2f); it was larger than in the vapour phase (Fig. 2g) and it increased sublinearly with the vapour phase composition. This is because that surface diffusion length of In adatoms on the NW-sidewalls was longer than that of Ga adatoms. Simultaneously, As trimers on (111)B top surface suppressed incorporation of Ga atoms and preferentially incorporated In atoms under *T*_G_ because the growth rate of the InAs and GaAs NWs in selective-area epitaxy showed different dependences on the AsH_3_ pressure. As was seen in Fig. 1, *T*_G_ was increased with decreasing In in the vapour phase. At higher *T*_G_, surface diffusion process of In atoms was also suppressed by the enhanced desorption; thus, the In composition in the solid phase was close to the In/Ga ratio in the vapour phase.

## Discussions

### InGaAs NW/Ge heterojunction

**Figure 3:**
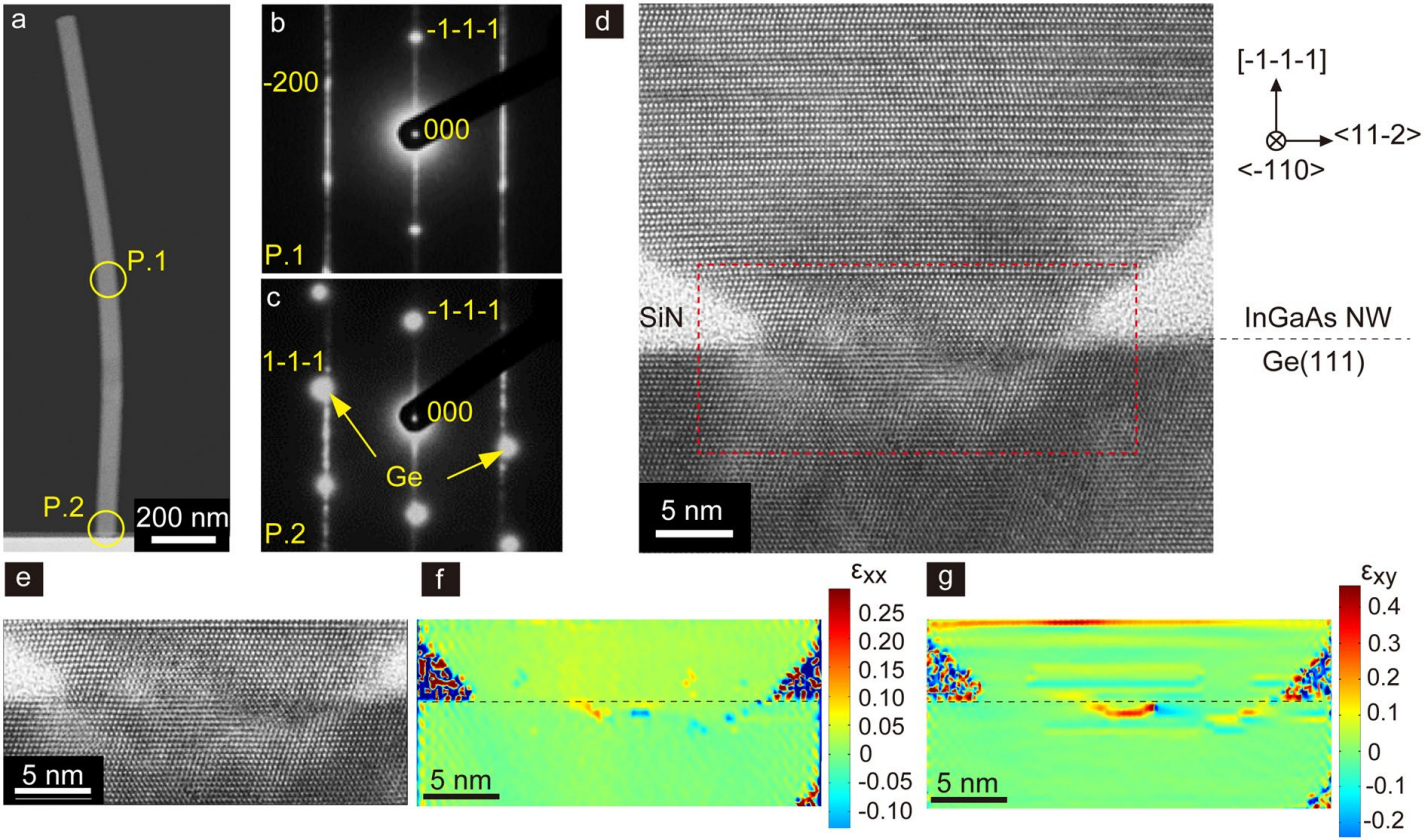
Crystal structure of the InxGa1 − xAs NWs on Ge and the InxGa1 − xAs NW/Ge heterojunction. (**a**) Low magnification TEM image showing In_0.68_Ga_0.32_As NW on p-Ge(111). (**b**) Selected-area diffraction (SAED) pattern of In0.68Ga0.32As NW denoted as P.1 in (**a,c**) Adjacent In_0.68_Ga_0.32_As NW/Ge denoted as P.2 in (**b**). (**d**) TEM image depicting the InGaAs NW/Ge(111). (**e**) Magnified TEM image of red dashed square in (**d**). Strain mappings estimated from the (**e**), (**f**) ɛ_xx_ mapping and (**g**) ɛ_xy_ mapping.

Figure 3 exhibits transmission electron microscopy (TEM) analysis of InGaAs NWs on Ge and the InGaAs NW/Ge heterojunction. In this case, the In composition of the solid phase was about 68%, for which the lattice mismatch was about 4.8%. Low-magnification TEM and selected-area electron diffraction (SAED) patterns indicated the InGaAs NW was directly integrated on Ge(111). Figure 3d shows the crystal structure in the vicinity of the InGaAs NW/Ge heterojunction. The heterojunction was 20 nm in diameter. The InGaAs NW grew epitaxially on the Ge substrate and had a zincblende (ZB) structure with many twinnings. The bottom of the NW, extending 5 nm from the NW/Ge heterojunction was pure ZB, then changed into a pure wurtzite (WZ) structure inside the SiN holes. Twinning was introduced above the SiN mask region.

As for the InGaAs NW/Ge heterojunction, the strain mappings in Fig. 3f,g show a local strain field originating from misfit dislocations (underneath the dashed lines). This indicated that the InGaAs NW slightly diffused into the Ge substrate and lattice relaxation with misfit dislocations occurred inside the Ge. The diffused InGaAs layer thickness was within about 1 nm of the junction. Importantly, only one dislocation formed across the InGaAs NW/Ge junction with the lattice mismatch of 4.8%. Conventionally, the period of the misfit dislocation network for the lattice mismatch would be about 68 Å, while in this NW system, the periodicity of the misfit dislocation network was broken and a lamellar strain field remained elongated in the plane. This was because a heteroepitaxial system close to being a coherent growth mode formed because of the small opening diameter. Similar behaviour was observed in selective-area epitaxy of GaAs NWs on Si^23^. An important point is that a similar coherent process is available when the diffusion of InGaAs occurs inside Ge and conventional crystal growth proceeds during the diffusion process. The average misfit dislocation and defect density (dangling bond density) was estimated to be about 3.2 × 10^11^ cm^−2^. The results are ascribed to that the InGaAs NW/Ge junction avoided the Bardeen limit^24^, meaning that the band alignment followed an anisotype heterojunction without Fermi-level pinning across the InGaAs/Ge junction^25^.

### InGaAs NW/Ge vertical tunnel diodes

**Figure 4:**
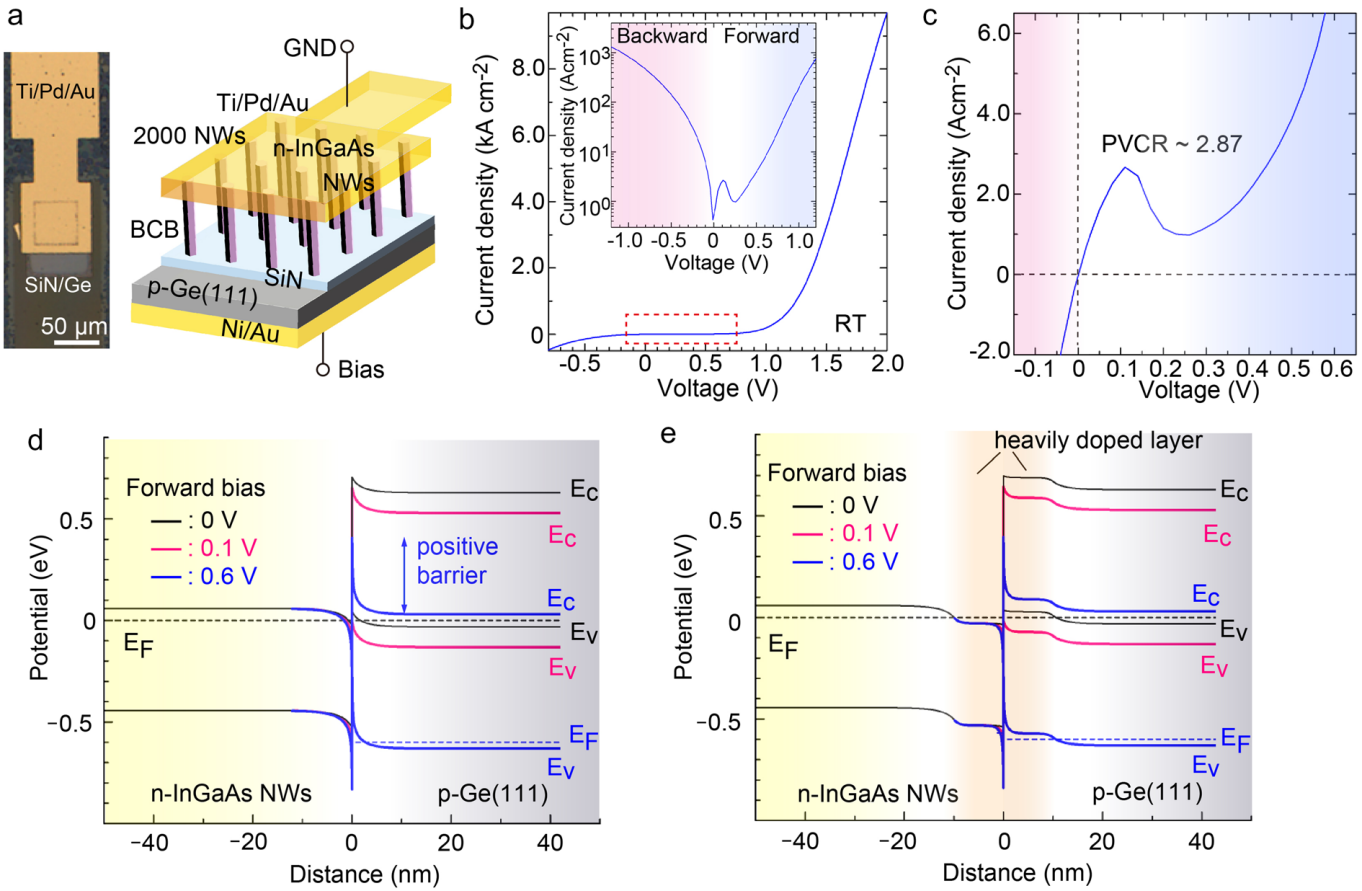
Diode properties using the vertical n-InGaAs NWs on p-Ge. (**a**) Illustration of In_0.8_Ga_0.2_As NWs/Ge vertical diode and plan view of optical microscopic image. 2000 NWs were connected to the top Ti/Pd/Au electrode. (**b**) Current density (J)–voltage (V) curve. Inset depicts the semi logarithmic plot. (**c**) Enlargement of the J–V curve of red dashed square in (**b**), (**d**) Band alignment for the n-In_0.8_Ga_0.2_As/p-Ge heterojunction simulated by one-dimensional Poisson–Schrodinger equation under various forward bias conditions. (**e**) Band alignment of the n-In_0.8_Ga_0.2_As/p-Ge heterojunction with heavily doped n^+^-InGaAs/p^+^-Ge layers in (**e**) under various forward bias condition.

A vertical diode composed of n-InGaAs NWs on p-Ge was fabricated, as shown in Fig. 4a. The composition of In atoms in the NW solid phase was 80% and NWs were doped with Si at a carrier concentration of 2.0 × 10^18^ cm^−3^ (The concentration was evaluated from the Si-doped InGaAs plane). The device region of the diode contained with 2000 NWs. The measured current was normalized by the contact surface area. The current density (J)–voltage (V) curve in Fig. 4b shows a rectification property with two turn-on voltages. One is the premature turn-on voltage of about 0.76 V which corresponded to the conduction band offset (ΔE_C_ ~ 0.75 eV), and the other is about 1.2 V which was much higher than ΔE_C_. A large turn-on voltage was estimated by extrapolating from the exponential region in the forward bias. The ideality factor and the series resistance were estimated to as 4.75 and 447 Ω, respectively. Interestingly, the J–V curve and its semilogarithmic plot (inset in Fig. 4b) exhibited the NDR at the peak voltage of 0.11 V in the forward bias direction. Figure 4c shows an enlargement of the red dashed square in Fig. 4b. The peak current density was 2.57 A/cm^2^ and valley current was 0.90 A/cm^2^. The peak-to-valley-ratio (PVCR) was 2.87. The peak current density was 2.8 A/cm^2^ at 0.12 V.

A simple n-InGaAs/p-Ge anisotype junction was numerically simulated (Fig. 4d) to compare with the situation in a thermal equivalent state (forward bias = 0 V) and the situation in which a forward bias is applied^26^. Under the thermal equivalent conditions, ΔE_C_ was estimated to be 0.65 eV, which corresponds to the built-in potential. ΔE_C_ was derived from the difference between work functions of the InGaAs NW and Ge, but ΔE_C_ can not explain the large turn-on voltage. When the forward bias was applied by ΔE_C_ (about 0.6 V), the positive barrier potential whose height of about 0.4 eV was formed as shown in Fig. 4d. Thus, the turn-on voltage of the J–V curve became larger than ΔE_C_. However, the simple band alignment depicted in Fig. 4e cannot explain the NDR phenomenon at a forward bias of 0.11 V.

Figure 4e simulates the band alignment of a InGaAs NW/Ge junction by introducing the heavily-doped n^+^-InGaAs and p^+^-Ge layers shown in Fig. 4d. Here, the carrier concentrations for the n^+^-InGaAs and p^+^-Ge were assumed to be 3.0 × 10^19^ cm^−3^, and the thickness of these layers was set to 10 nm. The band discontinuity had a large positive barrier at the forward bias of 0.6 V, which corresponds to a large turn-on voltage. Importantly, the anisotype heterojunction with the heavily doped layers formed a broken gap between the conduction band of the InGaAs NW and the valence band of Ge of 0.1 eV. At an applied forward bias of 0.1 V, the conduction band for InGaAs was overlapped with the valence band of Ge and Esaki tunnelling occurred. As the applied voltage was increased, the overlap disappeared and dissociation between bands occurred. Then, an NDR region appeared at around 0.11 V. The large turn-on voltage and the NDR shown in Fig. 4b can be reasonably explained by heavily doped InGaAs and Ge layers were naturally formed during the integration of the NWs on Ge.

The heavily doped layers are thought to originate from interdiffusion of the InGaAs and Ge substrate. As described in Fig. 3, the InGaAs NW diffused into Ge substrate. In this process, a large amount of In, Ga, and As was incorporated in the Ge as dopants from the InGaAs/Ge junction. This process was solid-phase diffusion. In the case of GaAs and GaN epitaxy on Ge substrates, Ga and As diffused into the Ge substrate and Ge outer diffused into the epilayers^27, 28^. Although the diffusion coefficient of As atoms in Ge is larger than the that of In and Ga^29–31^, the In and Ga atoms were heavily doped with As atoms in the vicinity of the n-InGaAs NW/p-Ge junction^27^. Additionally, the As dopant distribution deep in the Ge substrate would be compensated with substrate dopants (Ga dopants). By compensating the In, Ga, and As with the substrate impurities, a very thin Ge layer adjacent to the InGaAs/Ge junction would change into a p^+^-Ge layer.

While the thin InGaAs NW layer (including the NW layer underneath the junction) would be doped with Ge atoms and form a heavily doped n^+^-InGaAs layer in the vicinity of the junction. Ge atoms possess the amphoteric doping phenomenon. In the case of GaAs NW growth on Ge, the GaAs NWs were doped with a huge number of Ge atoms and showed photoluminescence related with Ge impurities and Ga vacancies^19^. Thus, the Ge atoms that outer diffused into the NW acted as n-type dopants. Thus, the thin n^+^-InGaAs layer was formed inside the NWs. When the InGaAs NWs were doped with Zn atoms by using the pulse doping technique^32^, the sharp NDR in the diode properties was vanished (Supporting Information Fig. S3). Importantly, a unique Esaki tunnel junction with high current density can be naturally formed through very simple direct integration of the InGaAs NWs on Ge regardless of the heavily doping and chemical doping^33, 34^. Similarly, n-GaAs NWs grown on p-Ge substrate showed small NDR signal (Supporting Information Fig. S5). The weak signal seemed to be the formation of p-GaAs buffer layer resulted in amphoteric doping^19^. While in case of InAs NWs on Ge, there was no NDR signal^15^. Thus, growth temperature for inducing interdiffusion and amphoteric doping behaviour strongly affect to the formation of the tunnel junction.

### Outlook

The findings in this research showing successful integration of vertical InGaAs nanowire array on germanium and creation of a functional material based on InGaAs/Ge heterostructures go against the common understanding in epitaxy and device physics that heterostructures in a lattice mismatched system always suffered from defects and a tunnel junction can only be realized by performing conventional doping in materials. As compared to the NW-based tunnel junctions^35–40^, the InGaAs/Ge tunnel junction shows the same level of PVCR. Thus, this study offers an alternative approach to creating a functional material system in unexplored combination without precise doping. As well as applications for high-performance transistors with co-integration of the InGaAs/Ge and tandem solar cells, high dense vertical tunnel diode array using the nanowire materials could pave the way to an unexplored circuit application for the Ge(111) on a Ge(111)-in-insulator substrates.

## Methods

### Selective-area growth for InGaAs NWs on Ge

The Ge(111) was Ga-doped p-type substrate with a carrier concentration of 3 × 10^18^ cm^−3^. 20-nm-thick SiN film was deposited by plasma-enhanced chemical vapour deposition (PECVD). Then, openings, 30 nm in diameter, were formed by electron-beam lithography and dry/wet etching. The InGaAs NWs were grown by metal–organic vapor phase epitaxy (MOVPE) with hydrogen (H_2_) carrier gas. Trimethylgallium (TMGa), trimethylindium (TMIn), and arsine (AsH_3_) gas were used as precursors. Mono-silane (SiH_4_) was used as n-type dopants. The initial Ge(111) surface changed into a (111)B-polar surface. The InGaAs NWs were grown at 670 °C using In compositions in the vapour phase ranging from 18 to 60% with a constant V/III ratio of 88. The partial pressure for the TMGa ([TMGa]) was ranged from 5.70 × 10^–7^ to 1.15 × 10^–6^ atm, and that of TMIn ([TMIn]) was ranged from 2.58 × 10^–7^ to 8.51 × 10^–7^ atm. The partial pressure of the AsH_3_ ([AsH_3_]) was 1.25 × 10^–4^ atm and of the SiH_4_ ([SiH_4_]) was 2.50 × 10^–8^ atm. As for the NWs for diode device, the NWs were doped with Si. In this case, [TMGa], [TMIn], [AsH_3_], and [SiH4] was 1.07 × 10^–6^ atm, 3.50 × 10^–7^ atm, 1.25 × 10^–4^ atm, and 2.50 × 10^–8^ atm. The *T*_G_ was 670 °C.

### Strain mapping

Strain mappings were calculated from the displacements of bright spots in Fig. 3e by using a peak-pair finding algorithm and the displacements of the bright spots were defined as u_xx_ = Δx − a_Ge(x)_ for the in-plane < 2–1–1 > direction and u_yy_ = Δy − a_Ge(y)_ for the vertical < 111 > direction^41^. Δx and Δy are the displacements of the bright spots in each direction, and a_Ge(x)_ and a_Ge(y)_ correspond to the lattice constants in the in-plane and vertical directions of the Si(111) substrate, as estimated from the TEM image. The strains ε_xx_ and ε_xy_ were determined as ε_xx_ = ${{\partial u} \mathord{\left/ {\vphantom {{\partial u} {\partial x}}} \right. \kern-\nulldelimiterspace} {\partial x}}$ and ε_xy_ = ${{\partial u} \mathord{\left/ {\vphantom {{\partial u} {\partial y}}} \right. \kern-\nulldelimiterspace} {\partial y}}$, where u is $\sqrt {u_{xx}^{2} + u_{yy}^{2} }$. Note that, since the displacement of the atoms was calculated using the positions of the atoms in crystalline Ge, InGaAs was mapped into a layer with a strain of + 4.8% by definition. The error in the strain calculation was approximately ± 0.5%.

### Device process for vertical NW diode

A two terminal device was fabricated for characterizing the electrical properties of the InGaAs NW/Ge interface by first coating the NWs with benzocyclobutene (BCB) by spin coating. Then, 30-nm-long of InGaAs NWs were revealed by reactive-ion etching using O_2_/CF_4_ mixed gas. Next, a 10-nm-thick Ti/10-nm-thick Pd/50-nm-thick Au multilayer was evaporated on top of the Sn-pulse doped InGaAs NW, and a 15-nm-thick Ni/50-nm-thick Au was deposited on p-Ge substrates. The devices were annealed at 250 °C for 3 min in N_2_.

### Numerical simulation for band alignment

Band alignment for the InGaAs/Ge heterojunction were calculated by using a one dimensional solar cell simulation (SCAPS-1D) program^42^.

## Supplementary Information

Supplementary Information.
