# Supplementary material for: Creation of unexplored tunnel junction by heterogeneous integration of InGaAs nanowires on germanium
Source: Sci Rep. 2022 Jan 31;12:1606. doi: 10.1038/s41598-022-05721-x (PMC8803860; doi:10.1038/s41598-022-05721-x)
Supplement: Supplementary file 1 — Supplementary Information. [file 41598_2022_5721_MOESM1_ESM.docx]

**Creation of unexplored tunnel junction by heterogeneous integration of InGaAs nanowires on germanium**

Akinobu Yoshida, Hironori Gamo, Junichi Motohisa, Katsuhiro Tomioka*

**Supplementary Information**

**1. Formation of (111)B polar surface on Ge(111) for selective-area growth of InGaAs nanowires on Ge.**

Conventional III-V nanowires (NWs) tend to grow in the <111>B or <111>A directions. For example, InGaAs NWs preferentially grow in the <111>B direction, so vertically-aligned InGaAs NWs can be grown on a III-V(111)B substrate. On the III-V(111)A surface, the InAs NWs grow in three equivalent tilted <111>B directions. The <111> direction of group-IV semiconductors, on the other hand, does not show the polarities such as A and B. Thus, in III-V/Ge heteroepitaxy equivalent surface orientations and directions always occur on the Ge(111) surface and these equivalencies form anti-phase domains. Instead of the anti-phase domain formation, equivalent growth directions always occur for III-V NW/Ge integrations. That is, such III-V NWs on Ge(111) grow in vertical <111> and three equivalent tilted <111> directions at the same time. The differences result either from the co-existence of (111)A and B surfaces that are formed when Si is eliminated by a metal catalyst during VLS growth, or from termination of group-III or group-V atoms on the Ge(111) surface during selective-area growth. For rational design of NW applications taking advantage of geometries, we have to force such equivalent growth directions into the vertical <111> direction.

As shown in Figs. S1(a) and S1(b), once As-incorporating Ge^3+^ and/or III-atom terminated Ge^1+^ has formed on the Ge surface, only vertical III-V NWs should grow on the Ge(111) substrates. This is because these surfaces are equivalent to a (111)B-oriented surface. Conversely, the growth directions of the III-V NWs can be controlled by optimizing the initial surface and growth conditions. To form an As-incorporated Ge^3+^ surface, group-V atoms should be replaced with the outermost Ge atoms of the 1×1 reconstructed surface because it is equivalent to a As-atoms terminated Ge^3+^ surface and a (111)B-oriented surface. The method of forming these (111)B-oriented surface is different for each III-V/Ge system because these processes depend on strength of the bond between group-III atoms and Ge atoms. Fig. S1(c) and S1(d) depict the growth sequence for aligning vertical InGaAs NWs on Ge(111).

The selective-area metal-organic vapour phase epitaxy (MOVPE) is illustrated in Figure S2. After the substrate was degreased [Fig. S2(a)], SiN films with thicknesses of 20 nm were formed by plasma-enhanced CVD (PECVD). Next, circular openings arranged in a triangular lattice with a pitch of 0.1 - 3.0 µm were formed on the SiN films by using electron-beam lithography and wet chemical etching. The opening diameter *d*_0_ can be adjusted by the lithography. In this paper, the *d*_0_ was ranged from 60 to 90 nm. Finally, InGaAs NWs were grown by MOVPE. The (111)B-oriented surface was used for the InGaAs NW growth because the III-V NWs are preferentially grown in <111>B directions. The (111)B surface has a topmost of the group-V atoms.


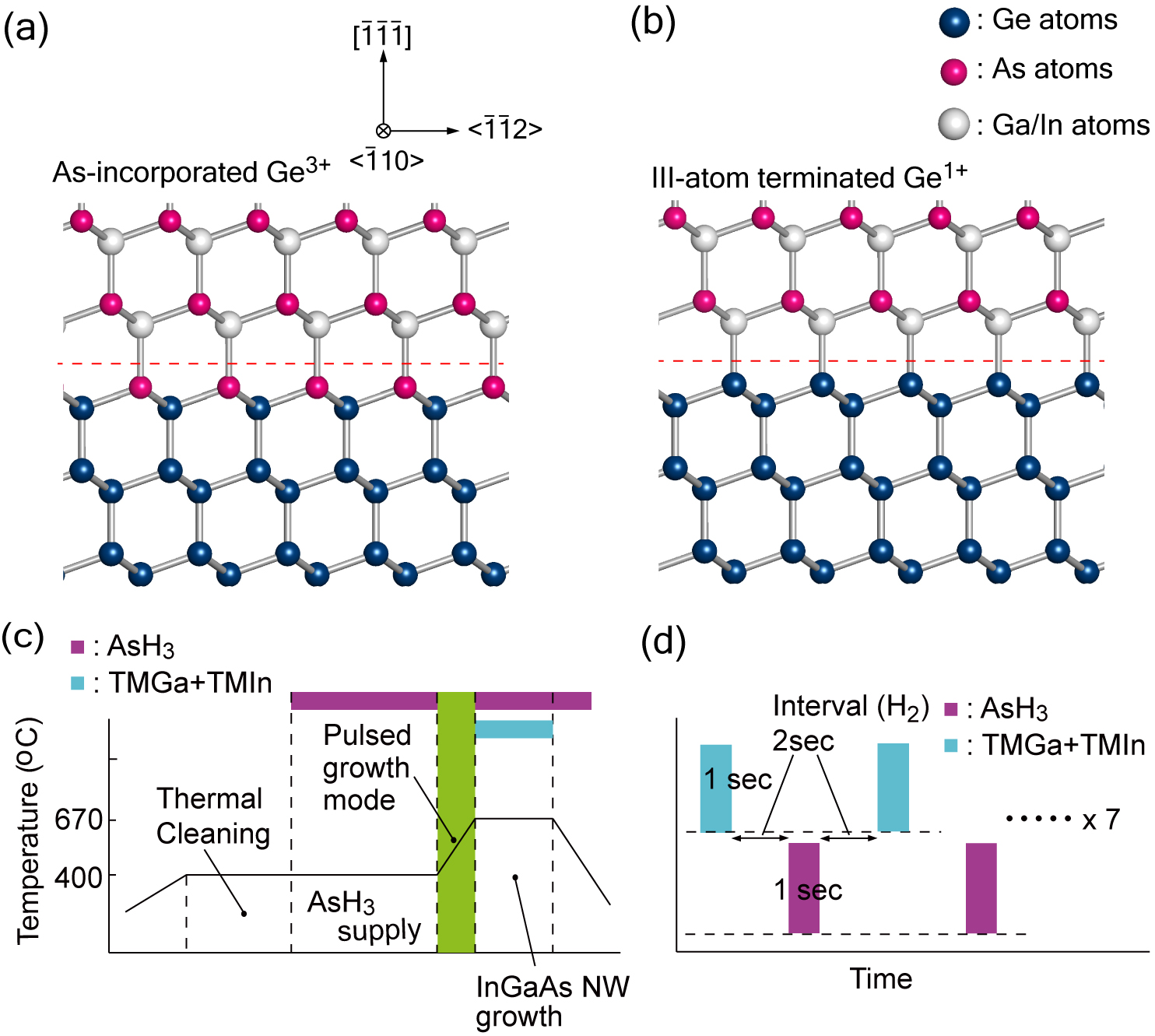


**Figure S1.** **Schematics of chemical structure**. (**a)** Group-V atoms incorporated Ge^3+^ surface, (**b)** Group-III atom terminated Ge^1+^ surface, (**c)** Growth sequence for aligning vertical InGaAs NWs on Ge. Thermal cleaning at 400°C in H_2_ is used to evaporate native oxide and form 1 × 1 surface reconstruction. AsH_3_ is supplied in order to form As-incorporated Ge^3+^ surface. The pulsed growth mode enhances the formation of III-atoms terminated Ge^1+^. (**d)** Schematic of pulsed growth mode. TMGa+TMIn (1 s) and AsH_3_ (1 s) are alternately supplied with an interval of H_2_ (2 s). This sequence is repeated 7 times.


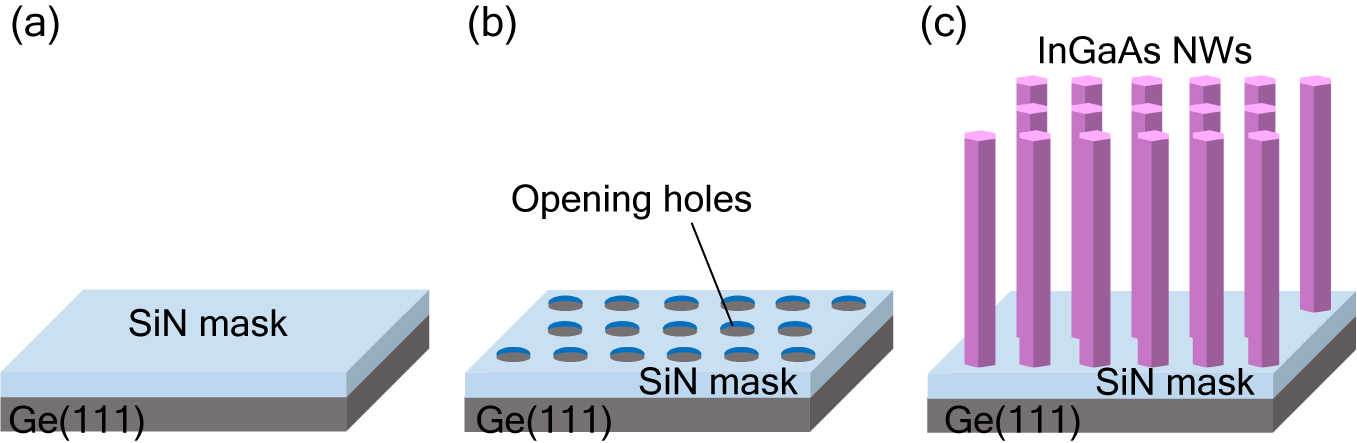


**Figure S2** Fabrication processes for selective-area MOVPE. (a) After the deposition of SiN film, (b) hole openings were formed by lithography and etching. (c) NWs were grown by metal-organic vapor phase epitaxy.

**2. Fabrication process for vertical NW diode structure.**

A two terminal device was fabricated for characterization of electrical properties in InGaAs NW/Ge interface by first coating the NWs with benzocycrobutene (BCB) by spin-coating [Fig. S3 (b)]. Then, 100-nm-length of InGaAs NW were revealed by reactive-ion etching with using O_2_/CF_4_ mixed gas [Fig. S3(c)]. Next, 10-nm-thick Ti/10-nm-thick Pd/50-nm-thick Au multilayer was evaporated on top of the Sn-pulse doped InGaAs NW [Fig. S3(d)], and 15-nm-thick Ni/50-nm-thick Au was deposited on p-Ge substrates. The devices were annealed at 250°C for 3 min in N_2_ [Fig. S3(e)].

Figure S3(f) shows the process results for Fig. S3(c) and Fig. S3(g) exhibits the optical microscopic image showing the plan-view of the device structure illustrated in Fig. S3(h).


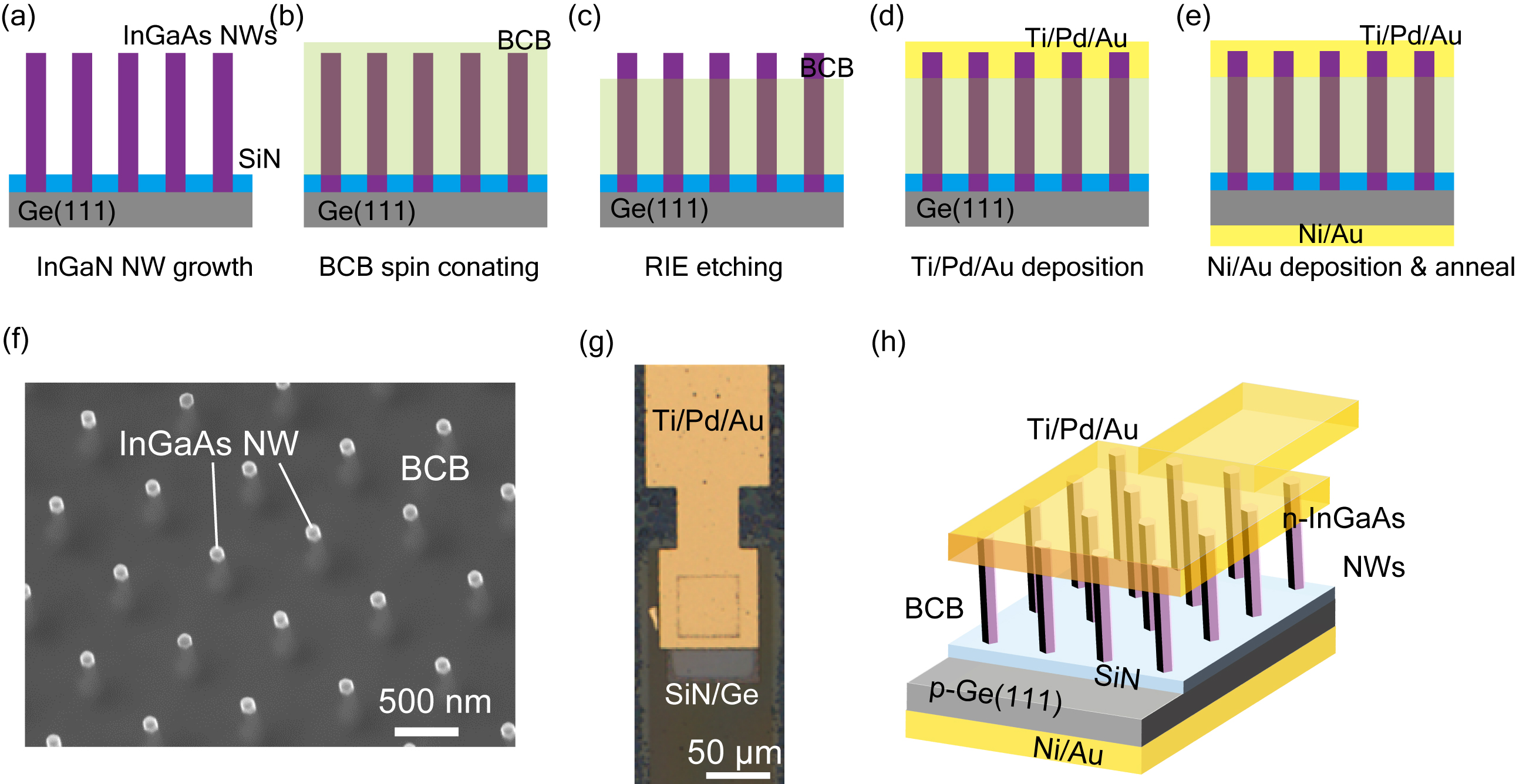


**Figure S3.** **Vertical NW diode structure**. (**a)** Selective-area growth of InGaAs NWs on Ge(111), (**b)** Spin-coating of BCB, (**c)** RIE process to reveal top portion of NWs. (**d)** Patterning by photolithography and evaporation of Ti/Pd/Au multi layers. **(e)** Deposition of Ni/Au and thermal annealing in N_2_. **(f)** SEM image showing the process result in panel **(c)**. **(g)** optical microscopic image showing the diode structure. **(h)** Illustration of vertical NW diode structure.

**3. The effect of forming Zn-pulse doped segment in InGaAs NWs for diode property.**

Figure S4 shows the current density (J) – voltage (V) curves for the vertical InGaAs NWs/Ge heterojunctions. The blue curve is measured from the NW array depicted in Fig. S4(b) which is same as the device shown in Fig. 4. Pink curve exhibits the NW array which has Zn-pulse doped segment in the vicinity of the InGaAs NW/Ge junction [illustrated in Fig. S4(c)]. Each device contains with 2000 NWs with the diameter of 40 nm (heterojunction is 20 nm in diameter).

When the Zn-pulse doped segment is formed inside the NWs, the NDR phenomenon due to the Esaki tunneling, originated from the heavy doping effect, is disappeared. This guarantees that the Esaki tunnel junction with unexplored InGaAs/Ge heterojunction was derived from the interdiffusion process and their heavy doping effect.


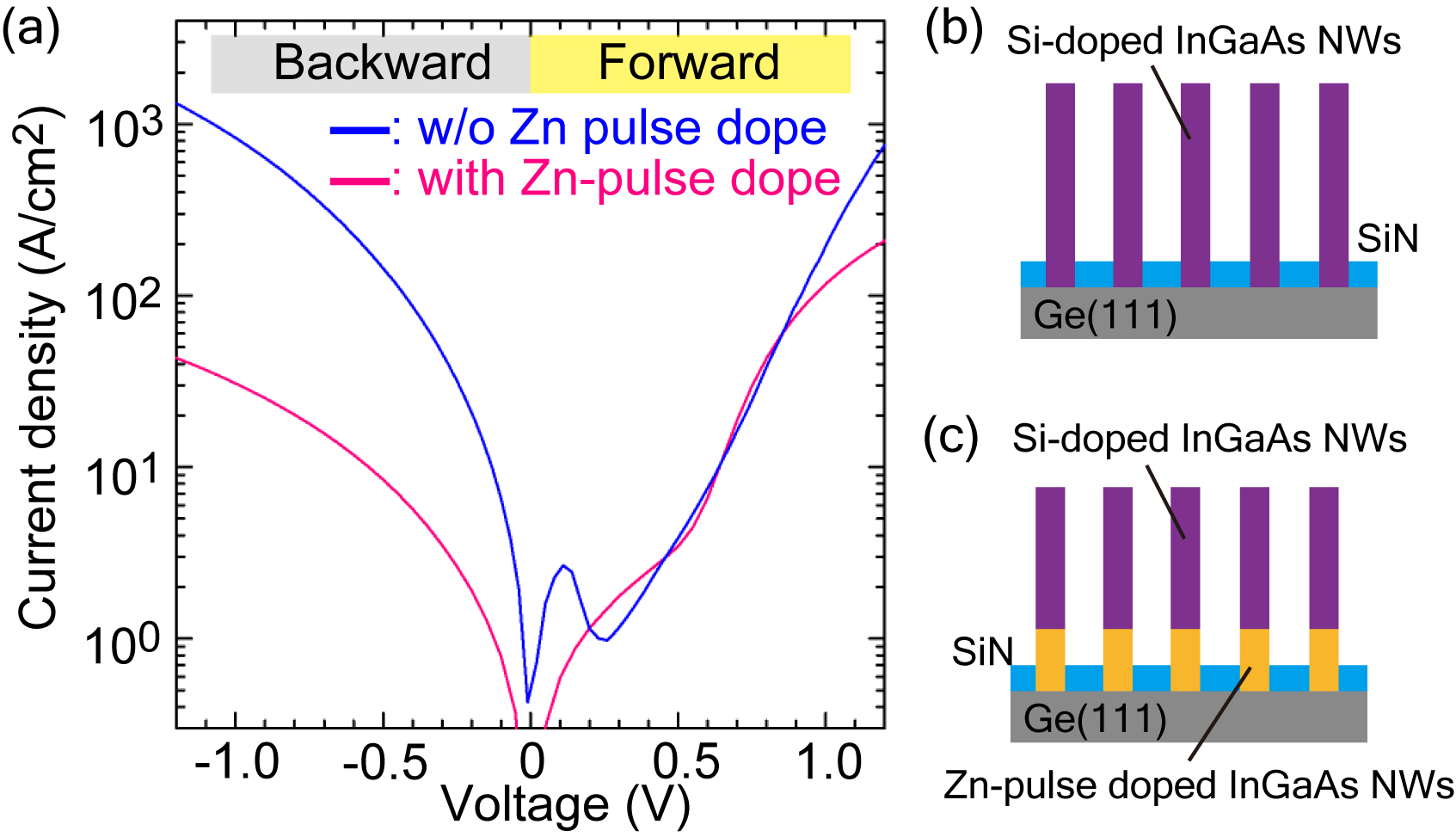


**Figure S4.** **Vertical NW diode properties with or without Zn-pulse doped segment in the InGaAs NWs**. (**a)** Semi-log plot for current density – voltage curve, (**b)** schematic structure of the Si-doped InGaAs NWs on p-Ge(111), (**c)** schematic structure of the Si-doped InGaAs NWs on p-Ge(111) with Zn-pulse doped segment.

**4. Si-doped GaAs NWs on Ge for diode property.**

Figure S5 shows the current density (J) – voltage (V) curves for the vertical n-GaAs NWs/p-Ge heterojunctions. The Fig. S5(b) shows small NDR signals in forward bias condition. In this growth, low-temperature buffer GaAs layer is grown to integrate the GaAs NWs on Ge. During the buffer growth, the Ge atoms ascribe to diffuse into Ga-sites in the buffer GaAs layer and the GaAs changed to p-type because of amphoteric behavior. And the Ge atoms was diffused into the GaAs layer as n-type dopant as similar to that of InGaAs NWs on Ge during the GaAs NW formation. As compared to the InGaAs NW growths, the carrier concentration of GaAs layer in the vicinity of the Ge substrate was lower than those of the InGaAs NWs on Ge. Thus, the NDR signal was weak in case of the GaAs NWs on Ge in Fig. S5.


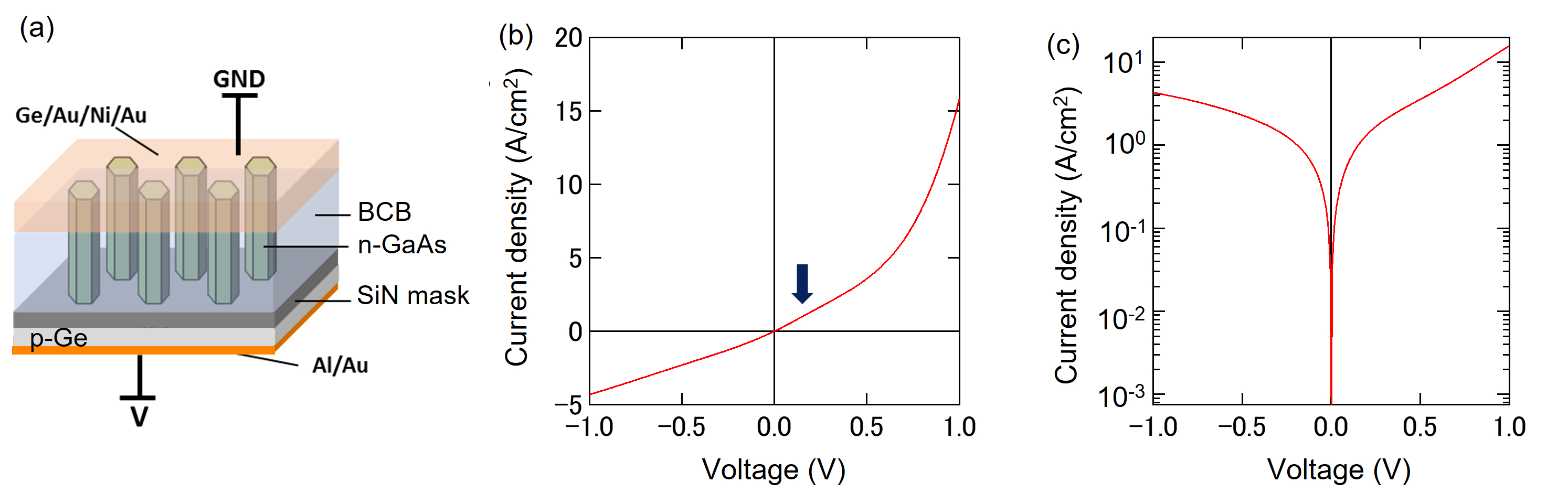


**Figure S5. Vertical NW diode properties using GaAs NWs on p-Ge.** (a) Illustration of vertical diode structure. (b) Current density – voltage curve. Black arrow shows NDR signal. (c) Semi-log plot of the panel (b).
